# Robust Radar Emitter Recognition Based on the Three-Dimensional Distribution Feature and Transfer Learning

**DOI:** 10.3390/s16030289

**Published:** 2016-02-25

**Authors:** Zhutian Yang, Wei Qiu, Hongjian Sun, Arumugam Nallanathan

**Affiliations:** 1 School of Electronics and Information Engineering, Harbin Institute of Technology, Harbin 150001, China; 2 State Key Laboratory of Urban Water Resources and Environment, Harbin Institute of Technology, Harbin 150001, China; qiuweihit@126.com; 3 School of Engineering and Computer Science, Durham University, Durham DH1 3LE, UK; hongjian.sun@durham.ac.uk; 4 Department of Informatics, King’s college London, London WC2R 2LS, UK; arumugam.nallanathan@kcl.ac.uk

**Keywords:** radar emitter recognition, Wigner–Ville distribution, three-dimensional distribution feature, transfer learning, relevance vector machine

## Abstract

Due to the increasing complexity of electromagnetic signals, there exists a significant challenge for radar emitter signal recognition. To address this challenge, multi-component radar emitter recognition under a complicated noise environment is studied in this paper. A novel radar emitter recognition approach based on the three-dimensional distribution feature and transfer learning is proposed. The cubic feature for the time-frequency-energy distribution is proposed to describe the intra-pulse modulation information of radar emitters. Furthermore, the feature is reconstructed by using transfer learning in order to obtain the robust feature against signal noise rate (SNR) variation. Last, but not the least, the relevance vector machine is used to classify radar emitter signals. Simulations demonstrate that the approach proposed in this paper has better performances in accuracy and robustness than existing approaches.

## 1. Introduction

Radar emitter recognition (RER) based on a collection of received radar signals is a specific type of identification termed specific emitter identification (SEI), which is used to distinguish different copies of the same type of radar emitter [1,2,3,4]. RER is a topic of wide interest in both military and civil applications [5,6]. In military applications, radar emitter recognition is a critical issue in radar reconnaissance systems [7]. Specifically, radar emitter recognition provides an important means of detecting hostile radar targets. In civilian applications, radar emitter recognition technology is used to suppress criminal activities by identifying navigation radars deployed on cars and ships [5]. However, the growing complexity of electromagnetic signals encountered in modern environments is a challenge for radar emitter signal recognition [7]. Traditional approaches are becoming inefficient against this emerging issue, especially when several radar emitter signals are captured [8,9]. Therefore, multi-component radar emitter recognition under a complicated noise environment is a challenge.

Many research works have been done, e.g., stochastic context-free grammar analysis [7,10], symbolic time series analysis [11,12,13] and time-frequency analysis [14,15,16,17,18,19]. Thereinto, time-frequency representation (TFR) has been frequently used for the analysis, the detection and the classification of nonstationary signals [20,21,22,23]. In [24], Swiercz proposed automatic classification of linear frequency modulation (LFM)signals for radar emitter recognition using wavelet decomposition and learning vector quantization (LVQ) classifier. In [25], a radar emitter recognition approach is proposed by using a classifier combining a minimum Mahalanobis distance classifier and SVM, which can recognize the numbers of intra-pulse modulation radar emitter signals. In [26], a hybrid radar emitter recognition is proposed. In [27], a robust radar emitter recognition based on fuzzy theory is proposed, which can recognize radar emitters robustly to some extent. However, these approaches have certain limitations for recognizing radar emitters in a complicated noise environment, which necessitates further investigation of robust radar emitter recognition.

Against this background, this research aims at proposing a robust scheme for multi-component radar emitter recognition under a complicated noise environment. The remainder of this paper is organized as follows. Section 2 gives the novel radar emitter recognition approach followed by the simulation and numerical analysis in Section 3. This paper is concluded in Section 4.

## 2. Radar Emitter Recognition System

### 2.1. System Model

**Figure 1:**
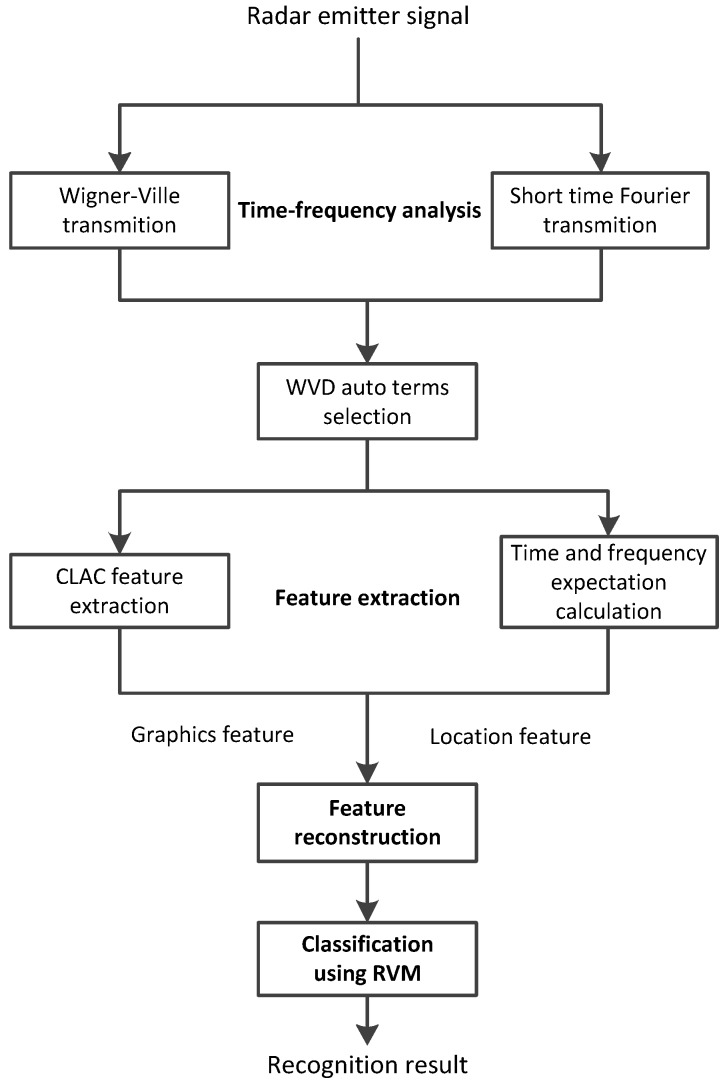
Model of the radar emitter recognition approach proposed in this paper.

The multi-component radar emitter recognition under complicated noise environment is studied in this paper. A radar emitter recognition approach based on the three-dimensional distribution feature and transfer learning are proposed. The process of radar emitter recognition is shown in Figure 1.

Three functional modules are included in this research:

1. time-frequency analysis,
2. feature extraction,
3. classification.

The time-frequency analysis is a preprocess of feature extraction and has been well known as a well-developed technique [28]. In this paper, the Wigner–Ville distribution (WVD) is used to represent the characteristics of radar emitters. However, the WVD cross terms are interferences of feature extraction. Therefore, the WVD cross terms are suppressed by using a superimposition of multiple spectrograms method [29]. The auto WVDs of radar signals are detected.

In the feature extraction block, a hybrid feature is extracted, which consists of a graphics feature and a location feature. Since redundancy features are contained in the graphics features, these features would be reconstructed. Feature reconstruction is based on transfer learning.

After sorting and feature extraction, radar emitter signals are described by vectors. These describing vectors are used for classification by using RVM.

### 2.2. Time-Frequency Analysis of Radar Emitter Signals

WVD is a famous time-frequency distribution method, which is widely used in signal processing for its good information-preserving property. The WVD of a given signal x(t) is defined by:

(1) $$W_{x}\left(t,f\right)=\int_{-\infty }^{+\infty }R_{x}\left(t,\tau \right)\exp\left(-2\pi jf\tau \right)d\tau$$

(2) $$R_{x}\left(t,\tau \right)=x\left(t+\frac{\tau }{2}\right)x^{*}\left(t-\frac{\tau }{2}\right)$$

where *τ* denotes the dummy variable; * and $W_{x}\left(t,f\right)$ denote the complex conjugate and the energy distribution of x(t) in frequency *f* at time *t*, respectively.

However, a criticism against WVD in application is the cross term, which is caused by the presence of several signal components. In order to suppress the cross terms caused by multi-component signals and noise, a suppression method by using the superimposition of multiple spectrograms is adopted. The spectrogram of the input signal can be obtained by the square of the short time Fourier transformation. Let f(t) be the input signal sampling value, namely f(t)=x(nΔt), where 0≤n<N. Therefore, in the case of the discrete signals, the short time Fourier transformation is given by:

(3) $$S_{f,L_{t},\beta }\left(n,k\right)=\sum_{m=0}^{N-1}f\left(m\right){g^{*}}_{L_{t},\beta }\left(m-n\right)\exp\left(-\frac{2\pi mk}{N}\right)$$

(4) $$g_{L_{t,\beta }}\left(n\right)=\frac{1}{\sqrt[{4}]{\pi L_{t}^{2}}}\exp\left\{-\frac{n^{2}}{2L_{t}^{2}}+j\frac{\beta }{2}n^{2}\right\}$$

where $g_{L_{i},\beta }\left(n\right)$ is a discrete window function, Lt=σtσtΔtΔt, $\beta =\eta \Delta t^{2}$.

The discrete signal spectrogram is given by:

(5) $$D_{f}\left(n,k\right)=\frac{1}{I+1}\sum_{i=0}^{I}{\left|S_{f,L_{i},\beta }\left(n,k\right)\right|}^{2}$$

Auto term regions of the spectrogram can be determined by judging $D_{f}\left(n,k\right)$ using a threshold, *i.e.*,

(6) $$\Omega =\left\{\left(n,k\right):D_{f}\left(n,k\right)\geq \eta \right\}$$

where *η* is the threshold, which is given by:

(7) $$\eta =\max\left\{\mu :\sum_{D_{f}\left(n,k\right)\geq \mu }D_{f}\left(n,k\right)\geq \gamma \sum_{n=0}^{N-1}{\left|f\left(n\right)\right|}^{2}\right\}$$

where *γ* is a factor, which means the ratio of the energy of the auto-term region against the whole WVD. It is proven by experiments that *γ* is between 0.7 and 0.98 [29,30], and we shall use 0.8 in this paper.

The auto-terms of WVD are obtained by determining regions, which is given by:

(8) $$\begin{array}{c} W_{auto}\left(n,k\right)=S_{\Omega }\left(n,k\right)W_{f}\left(n,k\right) \end{array}$$

(9) $$\begin{array}{c} S_{\Omega }\left(n,k\right)=\left\{\begin{array}{c} 1,\begin{array}{cc} & \left(n,k\right)\in \Omega \end{array}\\ 0,\begin{array}{cc} & \left(n,k\right)\notin \Omega \end{array} \end{array}\right\} \end{array}$$

Then, the auto-terms of WVD are obtained. The excellent mathematical properties of WVD are inherited. The expression of the interaction among the time, frequency and energy of a radar emitter is given in the form of WVD auto-terms. The auto-terms of WVD are normalized in each dimension and then used as the input of feature extraction. The approach to obtain the WVD auto-terms is stated with more details in [30].

### 2.3. Feature Extraction

The information of the distribution in dimensions of time, frequency and energy is an effective characteristic to distinguish different radar emitter signals. To extract the effective information in radar emitter signal pulses, a three-dimensional distribution feature is proposed, which consists of two parts, namely, the graphics feature and the location feature. This feature is used to extract information on WVDs of radar emitter targets.

The graphics feature is based on the cubic local auto-correlation (CLAC) function. The cubic local auto-correlation function is used for feature extraction and was proposed by Otsu in 2009 [31], which is an efficient way to describe three-dimensional distributions. The WVD-based CLAC feature is proposed and adopted as a graphics feature in this paper. A brief introduction of WVD-based CLAC feature is as follows.

Let g(r) be three-way (cubic) data defined on the region D:T×F×E with $r={\left(t,f,e\right)}^{T}$, where *F* and *E* denote the frequency and energy of the radar emitter signal, respectively, and *T* is the time length of the time window. Then, the *N*-th order auto-correlation function is defined by:

(10) $$\begin{array}{cc} & R_{N}\left(a_{1},\cdot \cdot \cdot ,a_{N}\right)=\int_{D_{S}}g\left(r\right)g\left(r+a_{1}\right)\cdot \cdot \cdot g\left(r+a_{N}\right)dr\\ & D_{S}=\left\{r|r+a_{i}\in D\forall i\right\} \end{array}$$

where $a_{i}\left(i=1,\cdot \cdot \cdot ,N\right)$ denote displacement vectors from the reference point r. We limit N≤2 and $a_{i}$ to a local region of a cube centering on r. A CLAC feature (vector) is made up of $R_{N}\left(a_{1},\cdot \cdot \cdot ,a_{N}\right)$ with various $a_{1},\cdot \cdot \cdot ,a_{N}$ in the local region. The computation of CLAC features is described as follows.

Firstly, Equation (10) is translated to a corresponding discrete version as Equation (11) shows,

(11) $$R_{N}\left(a_{1},...,a_{N}\right)=\sum_{t,f,e\in D_{S}}g\left(t,f,e\right)g\left(t+a_{1t},f+a_{1f},e+a_{1e}\right)\cdot \cdot \cdot g\left(t+a_{Nt},f+a_{Nf},e+a_{Ne}\right)$$

where $a_{ie},a_{if},a_{it}\in \left\{\pm \Delta r,0\right\}$ and N∈{0,1,2}, namely the same shifting distances are exploited in *t*, *f* and *e* dimensions. Then, the *N*-th-order autocorrelation function of binary images can be regarded as counting the number of points satisfying some logical condition, namely,

(12) $$g\left(r\right)\wedge g\left(r+a_{1}\right)\wedge \cdot \cdot \cdot \wedge g\left(r+a_{N}\right)=1$$

Therefore, the *N*-th-order autocorrelation function can be transformed into counting the patterns characterized by the above logical statement over *g*. Obviously, the scan by the reference point (t,f,e) can be restricted to the“1” points, *viz*., g(r)=1, in *g*. The configuration $\left(r,r+a_{1},\cdot \cdot \cdot ,r+a_{N}\right)$ is represented by a local mask pattern.

**Figure 2:**
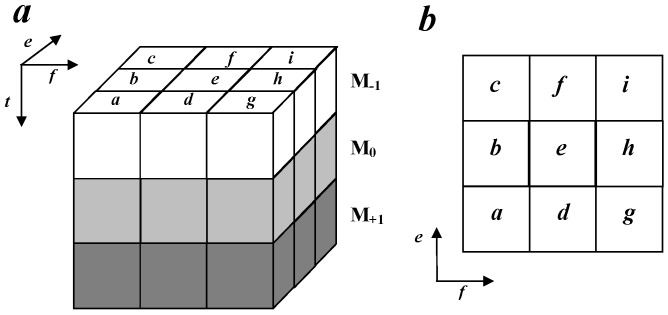
Model of the reference space.

Let ***M*** be the reference space insisting of 27 space units, as shown in Figure 2. In ***M***, there are three layers, namely $M_{-1},M_{0},M_{+1}$, and positions in each layer are termed a,b,c,d,e,f,g,h,i.

Each space unit in ***M*** is given a weight, which is $2^{n}$ $\left(n\in \left[0,1,\cdot \cdot \cdot ,26\right]\right)$. The convolution between ***M*** and the hyperplane of a WVD auto-term in the (*t*, *f*, *e*) space is carried out, which is given by:

(13) $$C\left(t,f,e\right)=\sum_{t}\sum_{f}\sum_{e}M\left(t^{\prime },f^{\prime },e^{\prime }\right)W\left(t-t^{\prime },f-f^{\prime },e-e^{\prime }\right)$$

where W(t,f,e) denotes the discrete WVD auto-term. Then, the number of each mask pattern in WVD is counted by comparing the convolution result matrix and flag values of mask patterns. The numbers of mask patterns are used as features.

Obviously, no more than three points can denote the *N*-th-order autocorrelation (N=0,1,2). There are many possible mask patterns, including duplicated patterns in terms of point configurations, and these mask patterns are reduced. In the case of binary image (f(r) = 0 or 1), $f{\left(r\right)}^{k}=f\left(r\right),\forall k>0$. The number of mask patterns is reduced to 251. Since the dimension of CLAC features corresponds to the number of mask patterns, we shall use the 251 dimensions features as the graphics features.

However, due to the shift-invariance of the autocorrelation, in the case that the point configuration of $\left(r^{\left(1\right)},r^{\left(1\right)}+a_{1}^{\left(1\right)},\cdot \cdot \cdot ,r^{\left(1\right)}+a_{N}^{\left(1\right)}\right)$ matches that of $\left(r^{\left(2\right)},r^{\left(2\right)}+a_{1}^{\left(2\right)},\cdot \cdot \cdot ,r^{\left(2\right)}+a_{N}^{\left(2\right)}\right)$ by shifting, $R_{N}\left(a_{1}^{\left(1\right)},\cdot \cdot \cdot ,a_{N}^{\left(1\right)}\right)$ will have the same value as $R_{N}\left(a_{1}^{\left(2\right)},\cdot \cdot \cdot ,a_{N}^{\left(2\right)}\right)$, which may lead to the confusion about the difference among targets with a similar WVD distribution. Therefore, positional information is adopted, so as to address the confusion caused by shifting in time and frequency. We locate the target WVD auto-term by computing the gravity center, which is represented by the time and frequency expectations. The time and frequency expectations of a WVD are given by:

(14) $${\hat{t}}_{\Omega }\left(t,f\right)=\frac{\int_{-\infty }^{+\infty }t^{\prime }\Omega \left(t^{\prime }-t,f^{\prime }-f\right)W_{x}\left(t^{\prime },f^{\prime }\right)dt^{\prime }df^{\prime }}{E_{\Omega }\left(t,f\right)}$$

(15) $${\hat{f}}_{\Omega }\left(t,f\right)=\frac{\int_{-\infty }^{+\infty }f^{\prime }\Omega \left(t^{\prime }-t,f^{\prime }-f\right)W_{x}\left(t^{\prime },f^{\prime }\right)dt^{\prime }df^{\prime }}{E_{\Omega }\left(t,f\right)}$$

where the $E_{\Omega }$ denotes the energy in region Ω.

In this paper, the gravity center position of the WVD auto-term is termed as the location feature.

### 2.4. Feature Learning Based on Transfer Learning

In order to characterize different radar emitter targets, the 3D feature is extracted. However, this feature has a very large number of dimensions, *i.e.*, 251 dimensions, which results in much redundant information for recognition. Therefore, the dimension reduction should be adopted. On the other hand, the variation of the signal noise rate (SNR) is an adverse effect on radar emitter recognition. Hence, a feature reconstruction approach based on transfer learning is adopted in this paper. By feature reconstruction, the robust feature against SNR variation is obtained, and the dimension of the feature is reduced, simultaneously.

Transfer learning is proposed to deal with the problem of how to reuse the knowledge learned previously from other data or features [32,33]. The idea behind transfer learning is to exploit the common knowledge between different learning tasks in order to share statistical strength and transfer knowledge across tasks [33,34,35]. In this paper, the learning task is to transfer common knowledge among recognitions in different noise environments.

**Figure 3:**
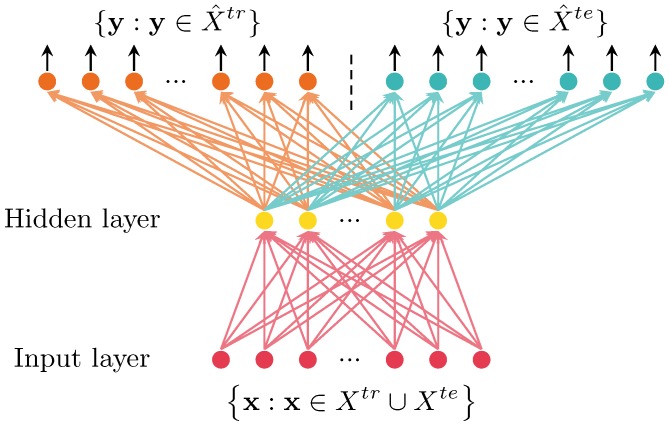
Illustration of the shared-hidden-layer autoencoder (SHLA) on the training set and test set.

The shared-hidden-layer autoencoder (SHLA) is an efficient approach for transfer learning [36]. It is utilized to obtain the compendious feature set with common knowledge from the original feature set. The structure of the SHLA is shown in Figure 3.

As shown, the target values are set to be equal to the input, and the hidden representation $h\left(x\right)$ is:

(16) $$h\left(x\right)=f\left(W_{1}x+b_{1}\right)$$

where f(z) is a non-linear activation function, f(z)=1/(1+exp(z)), $W_{1}$ is a weight matrix and $b_{1}$ is a bias vector.

The network output maps the hidden representation *h* back for the reconstruction of $\tilde{x}$:

(17) $$\tilde{x}=f\left(W_{2}h\left(x\right)+b_{2}\right)$$

where $W_{2}$ is a weight matrix and $b_{2}$ is a bias vector.

In SHLA, same parameters for the mapping are shared between the input layer and the hidden layer. However, independent parameters are used in the reconstruction process. Let $X_{tr}$ be the training set and $X_{te}$ be the test set. Two objective functions are given by:

(18) $$j_{tr}\left(\theta_{tr}\right)=\sum_{x\in \chi^{tr}}{∥x-\tilde{x}∥}^{2}$$

(19) $$j_{te}\left(\theta_{te}\right)=\sum_{x\in \chi^{te}}{∥x-\tilde{x}∥}^{2}$$

where the parameters $\theta_{tr}=\left\{W_{1},W_{2}^{tr},b_{1},b_{2}^{tr}\right\}$ and $\theta_{te}=\left\{W_{1},W_{2}^{te},b_{1},b_{2}^{te}\right\}$ share the same parameters $\left\{W_{1},b_{1}\right\}$.

Besides, the overall objective function is obtained by joining the two parts, given by:

(20) $$j_{SA}\left(\theta_{SA}\right)=j_{tr}\left(\theta_{tr}\right)+\gamma j_{te}\left(\theta_{te}\right)$$

where $\theta_{SA}=\left\{W_{1},W_{2}^{tr},W_{2}^{te},b_{1},b_{2}^{tr},b_{2}^{te}\right\}$, which will be optimized during training; *γ* is the hyper-parameter to control the strength of regularization.

In training, the back-propagation algorithm is adopted. The shared hidden layer makes the distribution induced by the training set similar to the distribution induced by the target set. Autoencoders find the useful features hidden in data automatically. This approach is wildly used in building a deep feature hierarchy, under the modelings of unsupervised, supervised and semi-supervised [37,38]. In this research, the reconstructed feature can be obtained from the hidden layer in SHLA for radar emitter robust recognition against SNR variation.

### 2.5. Classification Using RVM

The relevance vector machine (RVM), which is a sparse Bayesian modeling approach, is proposed by in [39,40]. RVM provides an approach for sparse classification. A small number of fixed basis functions are selected from a large potential candidate dictionary and weighted linearly. Therefore, a significant advantage of RVM is free from the satisfying Mercer’s condition of kernel functions.

For binary classification, the likelihood function is given by:

(21) $$P\left(t|\omega \right)=\prod_{n-1}^{N}\sigma {\left\{y\left(x_{n},\omega \right)\right\}}^{t_{n}}{\left[1-\sigma \left\{y\left(x_{n},\omega \right)\right\}\right]}^{1-t_{n}}$$

where $t_{n}\in \left(0,1\right)$ denotes the target value; ***ω*** is a Gauss conditional probability, which has zero expectation and variance $\alpha_{i}^{-1}$.

The maximum posterior probability estimation can be found by seeking the mode point of the Gaussian function, *i.e.*, $\mu_{MP}$.

Due to:

(22) $$P\left(\omega |t,\alpha \right)=\frac{P\left(t|\omega \right)P\left(\omega |\alpha \right)}{P\left(t|\alpha \right)}$$

the posterior probability according to ***ω*** can be maximized by maximizing:

(23) $$\begin{array}{c} \log\left\{P\left(\omega |t,\alpha \right)\right\}=\log\left\{P\left(t|\omega \right)\right\}+\log\left\{P\left(\omega |\alpha \right)\right\}-\log\left\{P\left(t|\alpha \right)\right\}\\ =\sum_{n=1}^{N}\left[t_{n}\log y_{n}+\left(1-t_{n}\right)\log\left(-y_{n}\right)\right]-\frac{1}{2}\omega^{T}A\omega +c \end{array}$$

where $y_{n}=\sigma \left\{y\left(x_{n},\omega \right)\right\}$ and *c* is a constant.

The marginal likelihood function is given by:

(24) Pω|t,α=∫Pt|ωPω|αdω≃Pt|ωMPPωMP|α2πMM22Σ1122

Suppose $\hat{t}$ is the approximation of the Gaussian posterior distribution, with $\mu_{MP}=\Sigma \Phi^{T}B\hat{t}$ and $\Sigma ={\left(\Phi^{T}B\Phi +A\right)}^{-1}$. Sparse Bayesian learning can then be formulated as a *type-II maximum likelihood* procedure. The logarithm of approximate marginal likelihood function is given by:

(25) $$\log p\left(t|\alpha \right)=-\frac{1}{2}\left\{N\log\left(2\pi \right)+\log\left|C\right|+{\hat{t}}^{T}C^{-1}\hat{t}\right\}$$

where $C=B+\Phi A^{-1}\Phi^{T}$.

Fast marginal likelihood maximization for sparse Bayesian models is exploited in this paper. More details of the fast approach can be found in [40].

## 3. Results and Discussion

The validity and robustness of the proposed approach will be evaluated by simulations. We investigate the performance of the proposed framework on an emulation dataset, which consists of six types of radar emitter targets generated emulationally based on the information of radars in the National Missile Defense (NMD) system [41]. In this dataset, mono-pulse radar and pulse compression radar are involved. The intra-pulse modulation types consist of linear frequency modulation (LFM), binary phase shift keying (BPSK) and quadri-phase shift keying (QPSK). The parameters of these radar emitter targets are given in detail by Table 1, where RF denotes radio frequency, PW denotes pulse width, FTR denotes frequency-time rate and CS coding scheme. The approaches proposed by Wang, L. *et al*. [27], Zhang, G. *et al.* [25] and Wu, Z. *et al.* [26] are for comparison. We note that the CLAC feature is adopted for these approaches in comparison.

Based on these signals, the training and test signals are generated respectively. Two hundred samples of each type radar emitter, 1200 in total, are used in training. In testing, the recognition accuracy is calculated averaged over 500 random generations of each type radar emitter. Both training and testing samples are mixed with additive white Gaussian noise. In order to evaluate the generalization performance of the recognition method, the training samples cover only samples of a 30-dB SNR, while the test samples cover samples in the range from −5 dB to 30 dB. In addition, the simulations were carried out on a personal computer with a 2.9-GHz dual-processor and 4 GB of memory.

**Figure 4:**
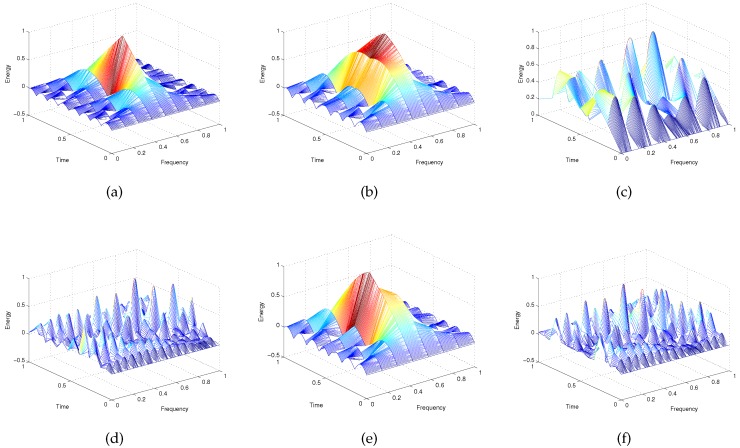
Normalized Wigner–Ville distribution (WVD) auto-terms of known radar emitter signals. (**a**) WVD auto-term of type 1 radar emitter signal; (**b**) WVD auto-term of type 2 radar emitter signal; (**c**) WVD auto-term of type 3 radar emitter signal; (**d**) WVD auto-term of type 4 radar emitter signal; (**e**) WVD auto-term of type 5 radar emitter signal; (**f**) WVD auto-term of type 6 radar emitter signal.

We evaluate the results of the radar emitter signal preprocess in Figure 4. Examples for radar emitter target distributions in the time-frequency-energy space are shown. We can see that WVD cross terms have been removed by using the superimposition of multiple spectrograms and threshold judgment. Each radar target is represented by its WVD auto-term. Furthermore, the relevance among time, frequency and energy is reflected exactly by the silhouette of the WVD auto-term, which will be the basis of the three-dimensional distribution feature extraction.

**Figure 5:**
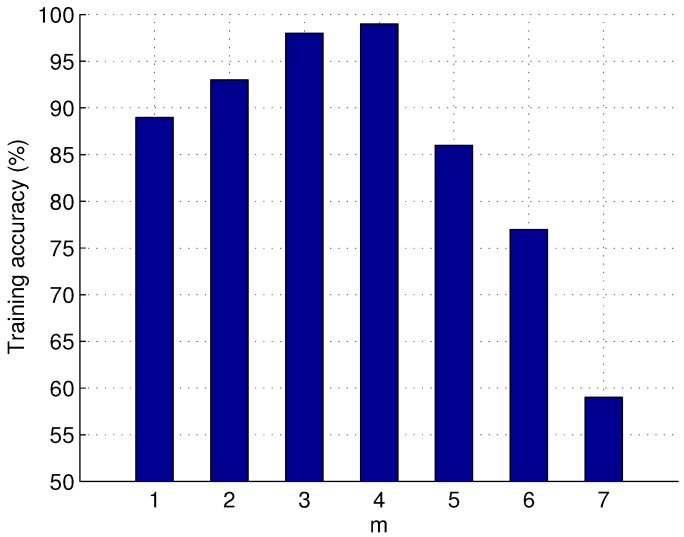
Training accuracy *vs.* *m*.

However, the silhouette of a radar emitter target WVD auto-term varies as the value of Δr varies. Different values of Δr will even lead to a different feature set. Therefore, we evaluate the training accuracy of the proposed approach over different Δr. The result is shown in Figure 5. To simplify forthcoming expressions, we use the parameter *m* to indicate the value of Δr. It is defined by $m\;=\;\log_{2}\Delta r$. Figure 5 indicates that the training accuracy reaches the peak when m=4. When m<4, the training accuracy increases as Δr increases. When m>4, the training accuracy decreases quickly as Δr increases. This is because sampling to WVD auto-terms is too sparse to express the details of the intra-pulse characteristics of radar emitter targets.

**Figure 6:**
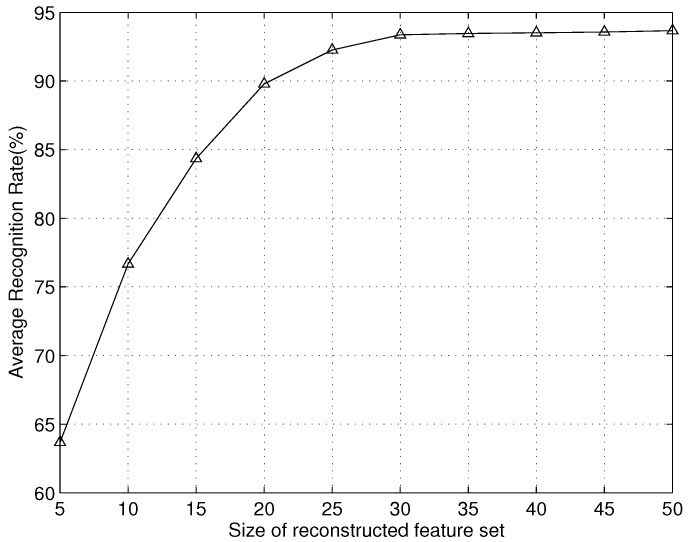
Recognition rate *vs.* size of reconstructed feature set *m*.

Next, we evaluate the performance of proposed scheme in term of the *average recognition rate* (ARR) over different sizes of the feature sets reconstructed, namely *m*. Actually, *m* is also the number of the hidden nodes of SHLA, which can influence the generation in different noise environments. In SHLA, the attempted hyper-parameter and weight decay values were the following: γ∈{0.1,0.3,0.5,1,2,3}, λ∈{0.0001,0.001,0.01,0.1}. As shown in Figure 6, the ARR increases as the size of the reconstructed feature set increases. When the size of the reconstructed feature set is 25, the training accuracy is more than 92%. Therefore, we can know that the proposed approach can get a satisfactory result to recognize the radar emitter targets with a small number of features. This result can prove the proposed approach’s validity well.

**Figure 7:**
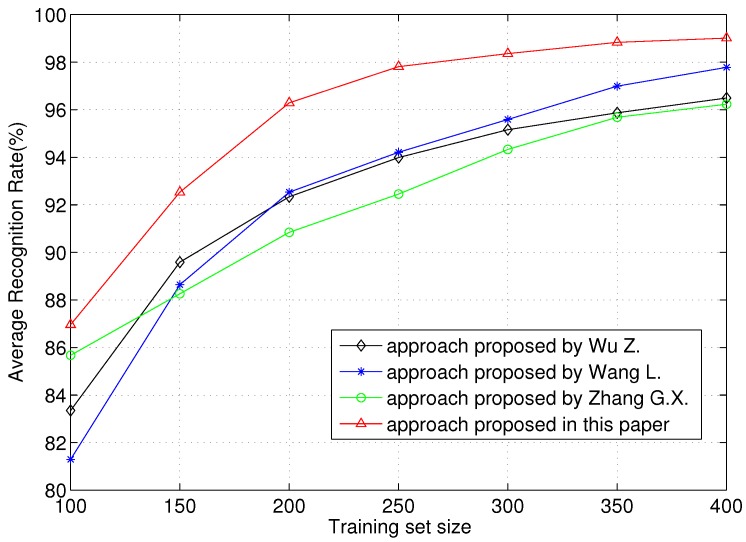
Average recognition rates *vs.* training set size.

In Figure 7, we evaluate the ARR performance of the proposed scheme over different training set sizes. We can know that the ARRs of all approaches in comparison increase as the training size increases. When the training sample number is more than 200, all approaches obtain more than 90% average recognition rates. In the comparison, the scheme proposed in this paper has the best performance. When only 100 samples are used for training, the ARR is more than 86%. When 300 samples are used for training, the ARR reaches more than 98%. This is because the WVD-based CLAC feature proposed in this paper has a high dimension and is efficient in representing the regularities among energy, time and frequency. The high dimensional feature has a good ability to reflect the details of samples, which is good for the following feature reconstruction. In addition, RVM is efficient at learning with a small size sample set. Therefore, the proposed scheme has a good performance in ARR over the training set size.

**Figure 8:**
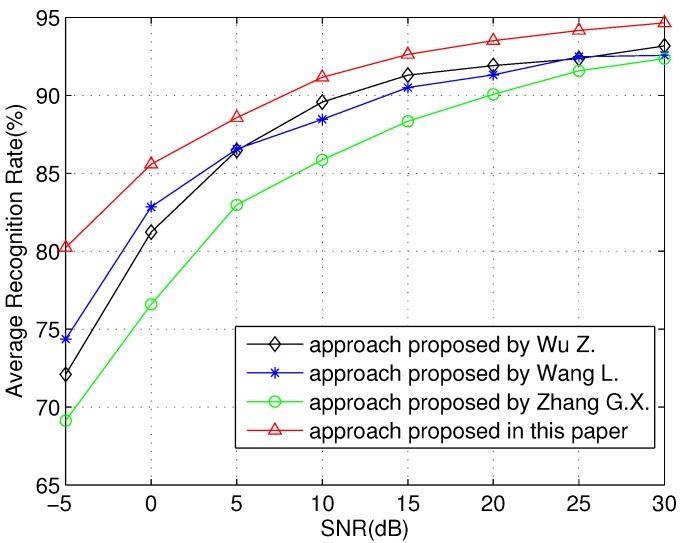
Recognition *vs.* signal to noise ratio.

In Figure 8, we evaluate the robustness performance of the proposed scheme over different SNRs. The proposed approach has a stable performance in different SNR conditions. In a low SNR environment, the proposed approach can recognize radar emitter targets effectively. When the SNR is -5 dB, the ARR is more than 80%, while those of other approaches are less than 75%. In a high SNR environment, the proposed approach has a high ARR. When SNR is 10 dB, the ARR approach proposed in this paper reaches more than 90%. This is because, during the feature reconstruction, the common knowledge among different SNR regions is found by using transfer learning. This knowledge makes the reconstructed feature robust against SNR variation. Moreover, the ARR of the approach proposed in this paper decreases gently as SNR decreases, unlike those of other approaches. Therefore, the result indicates that the approach proposed in this paper has robustness against SNR variation.

**Figure 9:**
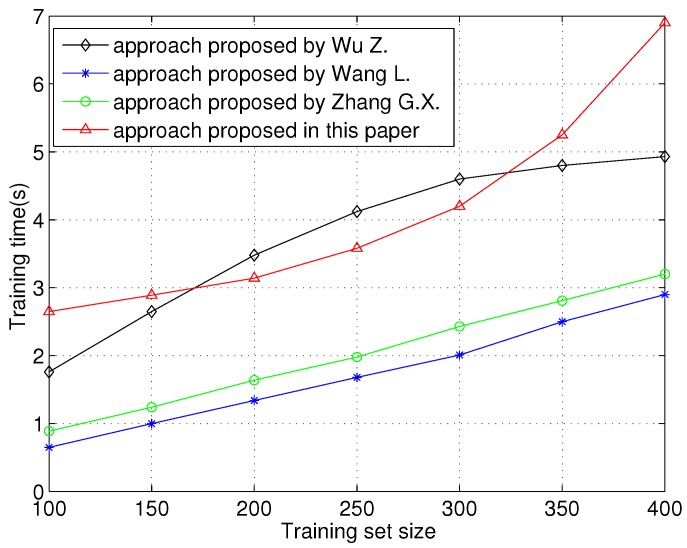
Training time *vs.* training set size.

Last, we evaluate the training time performance of the proposed scheme over different training set sizes. Because the same feature is used in all approaches, the time of feature reconstruction (feature selection) and classifier training is evaluated. As shown in Figure 9, approaches proposed by Wang L. and Zhang G.X. have good performances in training time. The approach proposed in this paper needs the most time for training. The training time increases rapidly as training set size increases. This is because SHLA used in this paper is a neural network-based transfer learning method. The high dimension of the CLAC feature goes against neural network training. Therefore, the training time performance of the proposed scheme is worse than other approaches.

## 4. Conclusions

In this paper, multi-component radar emitter recognition under a complicated noise environment is studied in this paper. A radar emitter recognition approach based on the three-dimensional distribution feature and transfer learning has been proposed, which can recognize the radar emitter robustly. Three functional modules are included in this approach, *i.e.*, time-frequency analysis, feature extraction and classification.

In preprocessing, the approach exploits the superimposition of multiple spectrograms represses the cross terms of WVD. In feature extraction, the three-dimensional distribution feature is proposed, which consists of two parts, *i.e.*, the graphics feature and the location feature. The three-dimensional distribution feature can represent the intra-pulse modulation information of radar emitter signals. In order to improve the robustness, the feature is reconstructed by using transfer learning, which can find the common knowledge in radar emitter signals in different noise environments. Therefore, the reconstructed feature is robust against SNR variations. RVM is applied to classify the radar emitter samples. It is proven by simulation that the proposed approach can recognize the radar emitter more accurately and robustly than existing methods. It is a new scheme for radar emitter recognition in complicated electromagnetic environments.

We admit that radar emitter recognition is subsequent processing of radar signal detection in an electronic reconnaissance system. The detection of the radar signal is not our focus in this paper. Improving training efficiency is the focus of our future works.

## Figures and Tables

**Table 1:** Information of radar emitter targets. PW, pulse width; FTR, frequency-time rate; CS, coding scheme; LFM, linear frequency modulation.

No.	Modulation	RF (MHz)	PW (μs)	FTR (MHz/μs)/CS
1	LFM	[4890, 5050], [5240, 5370], [5510, 5630]	[0.6, 1.2]	7.8
2	Mono-pulse	[5010, 5220], [5350, 5510]	[0.2, 0.5]	-
3	BPSK	[5260, 5550]	[0.3, 0.7]	Barker (7)
4	QPSK	[5410, 5510], [5630, 5680]	[0.6, 1.1]	Frank (16)
5	LFM	[5290, 5580]	[0.3, 0.6]	0.1
6	QPSK	[5500, 5620], [5660, 5730]	[1.0, 1.4]	Frank (16)
